# Neurodevelopmental effects of perinatal exposure to cannabis on progeny: A narrative review

**DOI:** 10.1016/j.dadr.2025.100372

**Published:** 2025-08-19

**Authors:** Chidimma Doris Azubuike, Oliver Grundmann, Amie J. Goodin

**Affiliations:** a Department of Pharmaceutical Outcomes and Policy, University of Florida, Gainesville, FL, United States; b Department of Medicinal Chemistry, University of Florida, Gainesville, FL, United States

**Keywords:** Cannabis, Neurodevelopmental effects, Prenatal, Perinatal, Pregnancy

## Abstract

**Objective:**

The certainty of effects on neurodevelopmental outcomes resulting from perinatal cannabis exposure is yet to be established. This review synthesizes current clinical and preclinical evidence on neurodevelopment and related functional outcomes in offspring exposed to cannabis during pregnancy or early childhood. Additionally, gaps in the literature and suggestions to bridge these gaps are provided.

**Method:**

A PubMed database search identified highest level of evidence studies focusing on *in utero* and early childhood cannabis exposure using keywords broadly describing outcomes alongside informal, spontaneous, and reference searches to supplement search hits. Priority was given to recent clinical studies. Findings were categorized into cognitive measures (memory, attention, and executive functioning), and diagnosis of mental health disorders (including: autism spectrum disorder [ASD], Attention deficit and hyperactivity disorder [ADHD], depression, anxiety, learning delays, and school-related performance). `

**Results:**

Findings on effects on cognition, autism, and learning are not consistent; however, compared to children who were unexposed, prenatally exposed children consistently have higher ADHD risk, and no significant association with anxiety and depression. Exposure to higher concentrations of tetrahydrocannabinol was found to be associated with more aggressive behavior in males compared to females.

**Conclusion:**

Most findings on perinatal cannabis exposure remain inconclusive. To enhance our understanding of associated neurodevelopmental effects, future research should reassess exposure over time, employ standardized cognitive measures, use reliable exposure assessments, and methods that consider cannabis concentration and composition across generations.

## Highlights

• Neurodevelopmental effects from perinatal cannabis exposure remain unclear.
• Prenatal cannabis exposure is linked to higher ADHD risk in children.
• Early cannabis exposure is associated with cognitive and behavioral deficits.
• Higher THC concentrations are associated with increased aggression in males.
• Future studies need standardized cognitive and reliable exposure measures.

## 1 Introduction

*In utero* exposures of cannabis may result in a variety of effects on the neurodevelopment of progeny, but the certainty of the effects and the severity of effects is yet to be established for many of these important outcomes, thereby creating uncertainty for clinicians and pregnant women who may consider the potential benefit of using cannabis but are uncertain about the potential associated risks (Cupo et al., 2024). In recent years, the use of cannabis has increased in the United States (Slawek et al., 2022). This trend is likely driven by greater accessibility due to state-level legalization of cannabis for medical and non-medical use (Avalos et al., 2024b, Bassalov et al., 2024, Gerede et al., 2024). Non-medical cannabis use, often described as recreational marijuana, has become available via programs in 23 states and some US territories, including the US Virgin Islands, Guam, the District of Columbia, and the Northern Mariana Islands (Haider et al., 2025). While medical marijuana programs are in various stages of implementation in 39 states and the District of Columbia as of 2025 (Haider et al., 2025).

The social acceptance and access to cannabis also appear to be increasing (Avalos et al., 2024b, Slawek et al., 2022). Therefore, health providers are likely to encounter patients using cannabis, whether under medical use contexts or not, including pregnant women (Bassalov et al., 2024, Slawek et al., 2022). Globally, the prevalence of cannabis use during pregnancy is between 1.4 % and 29.3 %, with the United States having the highest reported rates (Tadesse et al., 2024). This is likely influenced by the decriminalization of cannabis in developed countries such as the United States (Tadesse et al., 2024). Higher prevalence rates have been noted among younger pregnant women, specifically between the ages of 18–25 (Goodwin et al., 2020). Although cannabis and cannabinoid use during lactation, pregnancy, and preconception is cautioned against by the American Academy of Pediatrics and the American College of Obstetricians and Gynecologists (Bassalov et al., 2024), the rate of use continues to rise, with self-reports potentially underestimating use prevalence (Moore et al., 2023, Tadesse et al., 2024). For example, about 7–25 % of pregnant women (Chang et al., 2017, Moore et al., 2023) and about 12–20 % of children between ages 0–5 years tested positive for cannabis in populations sampled from urban medical centers (Moore et al., 2023, Sangmo et al., 2021).

Many women report perceiving cannabis to be safe for use while pregnant and report using it for nausea, mood, or recreational purposes (Bassalov et al., 2024, Cupo et al., 2024), so the potential for neurodevelopmental implications must be understood and risks appropriately communicated (Gerede et al., 2024). The lipophilic primary psychoactive substance (Δ9-tetrahydrocannabinol, THC) in cannabis that can cross the placental barrier, entering the bloodstream of the fetus, may potentially disrupt the neural development of the fetus (Avalos et al., 2024b, Bassalov et al., 2024). The endocannabinoid system, essential for the development of brain structures related to cognition, mood, and reward, is expressed at an early stage of fetal development in humans (Bassalov et al., 2024), therefore, such early exposures can potentially affect the formation of structures necessary for normal functioning. Many studies have examined the effect of perinatal cannabis exposure on the neurodevelopment of progeny, but findings so far remain challenging to disentangle, as some studies suggest negative effects, others positive effects, and some no effects at all. The current state of neurodevelopmental risks resulting from in utero cannabis exposures may leave clinicians and patients uncertain about the risks associated with the perinatal use of cannabis.

This review aims to synthesize current knowledge from clinical literature on neurodevelopmental and related functional outcomes in progeny who have been exposed to cannabis *in utero* or who have experienced secondary (“second-hand”) exposure to cannabis in early childhood. Additionally, this review considers what is known regarding the dose-response relationship associated with in utero cannabis exposures. This review also identifies existing gaps in the literature, and finally, we offer recommendations to bridge these gaps.

## 2 Methods

The study design is a narrative review. We constructed search strings to consider broad categories of neurodevelopmental outcomes (i.e., using keywords that described high-level outcomes rather than an exhaustive list of conditions and synonyms, as would be done for a systematic review), but limited the search for exposure to key terms and MeSH only for “cannabis”. To inform this review, an electronic search of the PubMed database was conducted in January 2025, alongside informal, spontaneous, and reference searches to supplement search hit findings. The following broad neurodevelopmental outcome categories were considered during searches: measures of cognition (including memory, attention, and executive functioning), and diagnosis of mental health disorders (including: autism spectrum disorder [ASD], Attention deficit and hyperactivity disorder [ADHD], depression, anxiety, learning delays, and school related performance).

Search results were filtered based on the following hierarchical criteria for prioritization of relevance to the study objectives. First, search result hits were prioritized for exposures of progeny to cannabis occurring in utero or occurring as secondary after birth in early childhood (e.g., ‘second hand’), and then for each neurodevelopmental outcome, where priority was granted to clinical studies. Next, inclusion in this review was prioritized for those studies demonstrating a high level of evidence on effects in humans, when available, such as meta-analysis and randomized clinical trials (RCTs). Lastly, we prioritized search results for studies by order of recency in cases where multiple studies of high quality were available for a given neurodevelopmental outcome. Results from studies are described and synthesized qualitatively, where primary findings are prioritized in their description and organized by neurodevelopmental outcome category. Analysis of potential risk of biases and gaps remaining were conducted following a qualitative description of studies for each outcome.

## 3 Results

Results are ordered by outcome category (see Table 1), where the outcome category described as measures of cognition appears first, followed by diagnosis of mental disorders as defined by the DSM-5 (American Psychiatric Association, 2013). While learning disorders are classified as neurodevelopmental outcomes under the DSM-5, those examined in this study were not assessed using formal DSM-5 criteria and are therefore considered applied neurodevelopmental outcomes in this study.

**Table 1:** Summary Characteristics of Included Studies.

**Author, Year**	**Study design**	**Sample size and/or total included studies**	**Study Details**	**Outcome measure**	**Key adjusted effect**	**95 % Confidence interval**	**Key findings/Conclusions**
**Measures of Cognition**							
**Memory**							
Gerede et al., (2024)	Review Study	Six studies	Assessment: Qualitative	Cognitive measure scales	Meta-analysis was not conducted	Meta-analysis was not conducted	Three studies showed no association with exposure to cannabis during pregnancy; others (3) found modest deficiencies in some cognitive measures, such as verbal comprehension, executive functioning, and attention
Torres et al., (2020)	Systematic Review	45 studies	Assessment: Qualitative	Cognitive measure scales	Meta-analysis was not conducted	Meta-analysis was not conducted	Prenatal cannabis exposure is not associated with lasting, clinically significant outcomes on cognitive function
Jarque et al., (2024)	Case Control	372 neonates	Country: Spain Exposure Assessment: Presence of cannabis in meconium samples Timing of exposure: 24 h following childbirth Follow-up: 36 months	Bayley Scales of Infant and Toddler Development (Bayley-III)	Exposed children's scores = 97.5 Unexposed children’s scores = 105	Exposed children's scores = 100–90 Unexposed children’s scores = 96.3–110 (p = 0.007)	Children exposed to cannabis during pregnancy had lower scores on the cognitive domain compared to unexposed children
Moore et al., (2023)	Cohort study	n = 1410	Country: United States Exposure measurement: Urine analysis Timing of exposure: 27 weeks of gestation and at 5 years of age Follow-up: Outcomes were assessed at age 5.	The NIH Toolbox Cognition Battery	Postnatal exposure: Difference in cognitive flexibility between exposed vs unexposed = - 15.6	−30 to −12	Lower cognitive flexibility was observed in children with early childhood exposure to cannabis compared to children who were not exposed Postnatal exposure to cannabis was also associated with more behavioral and cognitive problems compared to those unexposed. No difference was observed for prenatal exposure to cannabis
**Attention/Executive Functioning**							
Sorkhou et al., (2024)	Systematic review and meta-analysis	Six studies focused on attention (n = 12, 901, 376)	Assessment: Qualitative (Meta-analysis was conducted for other domains, but not attention.)	Attention scales	Meta-analysis was not conducted	Meta-analysis was not conducted	In early childhood and infancy, children who were exposed to cannabis during pregnancy were more likely to have poorer attention
Thompson et al., (2023)	Systematic review	Eleven studies (n = 523,107 patients)	Assessment: Pooled analysis	Attention scales	Standardized Mean Difference for attention (SMD) = −0.27	−0.60 – 0.07	Pooled standardized mean difference scores showed better performance for children who were not exposed to prenatal cannabis use compared to children who were exposed to cannabis use during pregnancy, but this finding was not significant
**Diagnosis of mental disorders**							
**ADHD**							
Tadesse et al., (2024)	Systematic review & Meta-analysis	14 studies (n = 203,783)	Assessment: Meta-analysis Ten studies on ADHD were included	ICD codes, CPT codes, and measurement scales.	β = 0.54	0.26 – 0.82	Children exposed to cannabis during pregnancy had a significantly increased risk of ADHD diagnosis
Bassalov et al., (2024)	Systematic review & Meta-analysis	18 studies (n = 534,445)	Assessment: Meta-analysis Six studies on ADHD were included	ICD codes and measurement scales.	OR = 1.13	1.01–1.26	Children exposed to cannabis in utero had higher odds of having ADHD diagnosis compared to unexposed children
**ASD**							
Tadesse et al., (2024)	Systematic review & Meta-analysis	14 studies (n = 203,783)	Assessment: Meta-analysis Four studies on ASD were included	ICD codes and DSM criteria.	RR = 1.30	1.03 – 1.64	Children prenatally exposed to cannabis had a significantly increased risk of ASD
Bassalov et al., (2024)	Systematic review & Meta-analysis	18 studies (n = 534,445)	Assessment: Meta-analysis Five studies on ASD were included	ICD codes and measurement scales.	OR = 1.04	0.79 – 2.29	Exposure to cannabis is not significantly associated with ASD development.
Avalos et al., (2024)	Cohort study	n = 178, 948	Country: United States Exposure measurement: Maternal self-report and positive toxicology test for THC in urine Timing of exposure: 8 – 10 weeks of gestation Follow-up: 143 months	ICD−9 and ICD−10 codes	HR = 1.05	0.84 – 1.32	No statistically significant association between children who were exposed to cannabis in early pregnancy and their counterparts who were unexposed
**Mood and Emotional Disorders**							
Bassalov et al., (2024)	Systematic review & Meta-analysis	18 studies (n = 534,445)	Assessment: Meta-analysis Five studies on anxiety were included	Anxiety questionnaires	OR = 0.72 0.11 – 4.57	0.11 – 4.57	Children exposed to cannabis during pregnancy were less likely to develop anxiety compared to children who were not exposed to cannabis use during pregnancy. However, the findings are not significant.
Bassalov et al., (2024)	Systematic review & Meta-analysis	18 studies (n = 534,445)	Assessment: Meta-analysis Three studies on depression were included	Depression questionnaire	OR = 1.34	0.79 – 2.29	Children exposed to cannabis during pregnancy had higher odds of developing depression compared to children who were not exposed to cannabis use during pregnancy. However, the findings are not significant.
Lepow et al., (2024)	Cohort study	n = 6146	Country: United States Exposure measurement: Self-report Timing of exposure: All stages of pregnancy Follow-up: 1 year	FMRI Task Behavioral scales: Child Behavior Checklist (CBCL), BIS/BAS (Behavioral Inhibition/Activation Systems), UPPS-P (impulsivity), Youth Prosocial Behavior Survey (PBS)	Superior frontal gyrus (L): −0.077	−0.13 to - 0.03	Children exposed to substance use, including cannabis, in pregnancy had differing brain activity accompanied by behavioral problems reported by their parents
Baranger et al., (2022)	Cohort study	n = 10632	Country: United States Exposure measurement: Self-report Timing of exposure: Exposure before and after maternal knowledge of pregnancy. Follow-up: 1-year & 2-year follow-up (Two cohorts included).	Child Behavior Checklist (CBCL), Prodromal Questionnaire Brief Child Version (PQ-BC).	Estimates were only displayed in a figure format. Estimates can be inferred from the figure	The confidence interval can be inferred from the figure.	Adolescents exposed to cannabis during pregnancy were more likely to experience psychopathological outcomes, including conduct problems, aggression, and rule-breaking behavior.
**Learning Delays**							
Avalos et al., (2024)	Cohort study	n = 119, 976	Country: United States Exposure measurement: Maternal self-report and positive toxicology test for cannabis in urine Timing of exposure: 8 – 10 weeks of gestation Follow-up: 5.5 years	ICD−9 and ICD−10 codes	Global delays: HR = 1.04 Motor delays: HR = 0.86 Speech and language disorders: HR = 0.93	Global delays: (0.68–1.59) Motor delays: (0.69–1.06) Speech and language disorders:(0.84–1.03)	No significant association between exposure to cannabis and neurodevelopmental delays
Talavera-Barber et al., (2023)	Cohort study	n = 138	Country: United States Exposure measurement: Self-report Timing of exposure: Latest use –Classified by trimester. First trimester (early), second, and third trimester (late). Follow-up: Outcome assessed at 12 months	Mullen Scales of Early Learning (MSEL)	Early vs none: Gross motor scores = 4.75 Late vs None: Expressive language scores = 6.48 Late vs none Receptive language scores = 3.67	Early vs none: Gross motor scores: (0.28 – 9.21) Late vs None: Expressive language scores: (2.05 – 10.91) Late vs none Receptive language scores: (0.08 – 7.26)	Infants exposed to cannabis performed better with higher scores for gross motor, expressive, and receptive language.
Jarque et al., (2024)	Cohort study	n = 64	Country: Spain Exposure Assessment: Presence of cannabis in meconium samples Timing of exposure: 24 h following childbirth Follow-up: 36 months	Bayley Scales of Infant and Toddler Development (Bayley-III)	Exposed children's scores = 92.5 (83.8–99.3) Unexposed children's scores = 97.0 (89.0–100.0)	Exposed children: (83.8–99.3) Unexposed children: (89.0–100.0)	Children exposed to any substance during pregnancy had an increased risk of language deficits compared to unexposed children
**School Performance**							
Gerede et al., (2024)	A review study	36 studies	Assessment: Qualitative Five studies on academic performance were included	Grade Point Average (GPA)	Meta-analysis was not conducted	Meta-analysis was not conducted	Exposure to cannabis in pregnancy is associated with poorer academic performance among exposed children compared to unexposed children
**Cannabis Associated Dose Response relationship**							
Aks et al., (2025)	Longitudinal study	n = 2262	Country: United States Exposure assessment self-report and hair toxicology analysis Timing of exposure: longitudinal over 4 years for cannabis users Follow-up: 48 months	Hair analysis for concentration of THC, CBD, and THCCOOH Child’s Behavior Checklist for externalizing and internalizing symptoms Exercise, asthma, and sleep (Munich Chronotype Questionnaire)	Correlation THC and exercise in males (coeff=5.31, p < 0.001) and CBD and sleep in males (coeff=1.98, p = .03) Correkation THC and sleep in females (coeff=−3.07, p < 0.01)		Differential effects of THC and CBD on physical and mental health (CBD calming, THC leading to aggression/activation) Sex specific differences, males stronger correlated exercise with increased THC concentrations Females negative correlation between increased THC levels and sleep quality
Wade et al., (2024)	Case control study	n = 246 (123 cannabis users matched with 123 non-users)	Country: United States Exposure assessment self-report and hair toxicology analysis Timing of exposure: longitudinal over 4 years for cannabis users Follow-up: 48 months	Hair analysis for concentration of THC, CBD, and THCCOOH Neurocognition: Picture Sequence Memory Test, Picture Vocabulary Test, List Sorting Working Memory Test, Flanker Inhibitory Control and Attention Test, Pattern Comparison Process Speed Test, and Oral Reading Test	Average hair concentrations: THC:42.7 pg/mg, CBD: 16.7 pg/mg THCCOOH: 6.2 pg/10 mg Picture memory: 105.8 vs. 111.0 (p = .03) Picture vocabulary: 101.8 vs. 105.2 (p = .07) Picture vocabulary & THCCOOH regression: r = −0.2, p = .03 Flanker & THCCOOH regression: r = −.19, p = .04	THC: 0–497 pg/mg CBD: 0–341 pg/mg THCCOOH: 0–103 pg/10 mg Picture memory: 68–146 Picture vocabulary: 70–146	Cannabis use was associated with lower scores on episodic memory tasks Correlation between cannabis use through confirmed THCCOOH hair analysis with lower neurocognitive performance
Avalos et al., (2024)	Cohort study	n = 22,624	Country: United States Exposure assessment: self-report and urine toxicology screen Timing of exposure: any cannabis use during pregnancy Follow-up: first post-partum care visit	Birthweight, gestational age, preterm birth	Low birth weight (OR:1.20) Small for gestational age (OR:1.24) Preterm birth (1.06)	Low birth weight 20 [1.12–1.27] Small for gestational age [1.18–1.30] Preterm birth [1.00–1.12]	Higher risk of low birth weight, small for gestational age, preterm birth, and NICU admission requires counseling of pregnant individuals to reduce adverse neonatal health outcomes.
Grant et al., (2018)	Review study	n = 50 studies	Countries: United States, Canada, Netherlands, France, United Kingdom Variable outcomes, most assessed birthweight and behavior close to birth, some evaluated cognition Follow-up: variable, from 1 to 22 years	Birthweight, cognition, attention	Reduced birthweight Reduced attention 18 months – 12 years after birth Reduced verbal memory processing		Fetal exposure may lead to reduced birthweight but does not impact long-term growth Global intelligence not affected but certain executive functioning is negatively impacted In utero cannabis exposure leads to higher rates of depressive and anxiety disorders in adolescence

### 3.1 Measures of cognition

Researchers in different fields have defined cognition in several ways. In this review, we will define cognition measures according to how they were defined in the reviewed studies.

#### 3.1.1 Memory

Findings on the effect of prenatal cannabis exposure on offspring’s cognition were largely varied. One review noted that differences in cognitive effects could be attributed to variations in the type of measurement instrument, study design approaches, and the variables controlled for in each study (Gerede et al., 2024). This review of six studies conducted between 2014 and 2023 found that while a few studies showed no association with exposure to cannabis during pregnancy, others (3) found modest deficiencies in some cognitive measures such as verbal comprehension, executive functioning, and attention.

Alternatively, a systematic review of 45 studies between 1988 and 2016 examining the cognitive impact of prenatal cannabis use on progeny found no significant clinical effects (Torres et al., 2020). Cognition in the included studies was assessed in a total of 1004 ways, including attention span, visual recognition memory, language development, general intelligence, goal-directed action, etc. Among these studies, 34 reported poorer cognitive outcomes in children prenatally exposed to cannabis, while 9 studies found better cognitive outcomes in exposed groups. The authors concluded that prenatal cannabis exposure was not associated with lasting clinically significant outcomes on cognitive function, citing that scores generally fell within the normal range. However, this conclusion may be overstated, as only a limited number of measures (3 %) were compared to normative benchmarks. This study attracted substantial attention, with two commentaries published in response to their findings.

The first commentary noted that the conclusions made by the authors were inappropriate because making such conclusions would require a meta-analysis, which the authors did not conduct. Additionally, the interpretation of the term “clinically significant” was deemed problematic because the authors based their judgments on the assertion that the scores were within the normative range. However, this commentary noted that the absence of clinical significance should not imply a lack of impact on patients' lives, especially in cases with persistent lower scores. Furthermore, the lack of substantial evidence should not be misconstrued as an indication of safety (Chaput et al., 2020). The authors also noted that about 80 % of the included studies were drawn from three longitudinal cohorts with cannabis use data collected between the years 1970 and 1980, and considering that the potency of cannabis has increased in recent years, it may be inappropriate to generalize conclusions to the contemporary context (Chaput et al., 2020).

The second commentary further criticized the inclusion of 10 studies, stating that their aims were focused on assessing the impact of prenatal cocaine use in offspring with cannabis included as a covariate, and the exclusion of a supposedly important study (Singer et al., 2021). Torres et al. (2021) responded to the second commentary stating that these 10 studies were included because, although the studies did not aim to examine the impact of prenatal cannabis exposure on cognitive outcomes, these studies drew conclusions regarding the effect of cannabis exposure on offspring and further stated that the excluded study did not assess cognitive functioning outcomes in offspring. The lack of a systematic quality assessment in the review by Torres et al. (2020) (for example, only 12 of the 45 included studies measured cannabis exposure through a urine test or meconium, and about 28 studies did not report mean scores of the cognitive outcomes assessed), and the lack of consensus in this area highlights the need for more rigorous research. Additionally, conclusions about cognition in general may be somewhat problematic because cognition is a multifaceted concept, comprised of several facets, including attention, memory, executive function, etc.

Although the studies conducted between 1970 and 1980 are older and involved cannabis concentrations that are lower compared to current concentrations, they represent some of the earliest studies on prenatal cannabis exposure (Thompson et al., 2023, Torres et al., 2020). These foundational studies explored the effect of cannabis on offspring exposed to cannabis during pregnancy. However, there are some limitations to be noted. Information on cannabis exposure was collected via maternal self-report, which limited accuracy (Torres et al., 2020). In some studies, where women included consumed other substances such as alcohol and tobacco, it was difficult to disentangle the effect of cannabis from other substances consumed (Torres et al., 2020).

In line with some of the findings from a previous review (Gerede et al., 2024), a case-control study of newborns delivered between 2018 and 2019 investigated the effect of substance use in children, in a cohort of children requiring admission to the Neonatal Intermediate and Intensive Care Unit (NIMCU and NICU) at 3 years old using the Bayley Scales of Infant and Toddler Development (Bayley-III), found a significant negative association (Jarque et al., 2024). This study examined the impact of any substance use during pregnancy measured in meconium samples and its subsequent effect on motor, cognitive, and language development. Results showed that children exposed to cannabis during pregnancy had lower scores on the cognitive domain compared to unexposed children (97.5 [100–90] vs. 105 [96.3–110]; p = 0.007]), with effects more common and severe among male offspring exposed to cannabis.

A few studies have examined the effect of prenatal and early childhood exposure to cannabis and cognitive outcomes in children. Children from a cohort of mothers between 2010 and 2014 who were postnatally examined for cannabis exposure measured in urine samples at the age of 5 were found to have more behavioral and cognitive problems compared to those unexposed (Moore et al., 2023). For example, less cognitive flexibility was observed in children with early childhood exposure to cannabis compared to children who were not exposed. Using the NIH toolbox task, a mean difference of −15.6 with a 95 % confidence interval of −30 to −1.2 was observed. However, no difference was observed for prenatal exposure to cannabis. The finding of decreased cognitive flexibility in postnatally exposed children reflects a similar effect to other studies that have found significant negative effects of prenatal cannabis exposure on offspring (Gerede et al., 2024, Jarque et al., 2024), suggesting that the impact of cannabis on offspring may be similar for both prenatal and postnatal exposures.

Similarly, a prospective cohort study of pregnant women recruited at the fourth and fifth months of pregnancy in years 1982–1985 investigated the effect of early use of marijuana on young adults adjusting for cannabis use during pregnancy (marijuana) (Willford et al., 2021). Findings showed that the use of cannabis (marijuana) in early stages of pregnancy, assessed through self-reports, indirectly influenced adult memory through its effect on childhood memory deficits. However, the direction of this effect was not explicitly stated, and the findings of this study may not be generalizable to the present. Although (Jarque et al., 2024) and (Moore et al., 2023) assessed cannabis exposure via urine analysis, suggesting more objective exposure assessment, the lack of consensus between these studies denotes a need for further confirmatory research.

#### 3.1.2 Attention/executive functioning

Attention has been investigated in several studies and has mainly been assessed using various behavioral and attention scale measures. A systematic review qualitatively synthesized evidence from studies published between 1999 and 2021 to investigate the influence of prenatal exposure to cannabis on outcomes such as attention, intelligence quotient (IQ), and memory, and found significant negative outcomes (Sorkhou et al., 2024). Of the 6 qualitatively examined studies on attention, all were rated high-quality according to the Newcastle Ottawa Quality Assessment Scale (NOS). Two studies on infants found a negative impact of cannabis on exposed infants compared to unexposed, including higher odds of attention problems in girls and decreased response to stimuli. Three out of four studies on toddlers found negative effects of cannabis in toddlers who were exposed to cannabis during pregnancy compared to unexposed. These studies measured attention based on several attention measurement scales, including the continuous performance test (CPT), Child Behavior Checklist (CBCL), etc. Revealing that in early childhood and infancy, children who were exposed to cannabis during pregnancy were more likely to have poorer attention.

In contrast, a previously conducted systematic review of studies published between 1991 and 2021, conducted to assess the impact of cannabis use in pregnancy and neurobehavioral effects on offspring between ages 6–18 years found no significant difference. (Thompson et al., 2023). Although they reported that of 11 studies included, 6 studies found a significant difference between attention scores, and 5 studies found no significant difference between attention scores, pooled standardized mean difference scores seemed to reflect better performance for children who were not exposed to prenatal cannabis use compared to children who were exposed to cannabis use during pregnancy, but this finding was not significant SMD = -0.27 [95 % CI: −0.60 – 0.07]. The Studies included in each systematic review and meta-analysis often represent a pooled analysis of studies conducted over an extensive time frame. It may be necessary to carry out a systematic review focusing solely on more recent literature for more definitive findings. For instance, in the studies assessed by Thompson et al. (2023), the six studies that identified significant effects were conducted between 1991 and 2003. In contrast, studies that found no significant differences were conducted between 2011 and 2021. These generational differences between time points may have skewed the overall pooled estimates from the meta-analysis. Consequently, updating the literature with more current research and meta-analysis in this area may be essential. Furthermore, we acknowledge that there is a large body of literature that assesses measures of cognition or indicators of potential cognition based on investigation of the development of physical structures in the brain or imaging assessments of growth trajectories or brain activity (Hiraoka et al., 2023). But we consider this research out of scope for our objective, as we are reviewing observable effects on behaviors in progeny for cognition.

### 3.2 Diagnosis of mental disorders

Mental health disorders such as autism spectrum disorder (ASD), Attention deficit and hyperactivity disorder (ADHD), depression, and anxiety are described below.

#### 3.2.1 Neurodevelopmental disorders

There has been a significant number of research investigating the link between cannabis exposure during pregnancy and the occurrence of ADHD diagnosis in progeny. A meta-analysis of ten cohort studies (Tadesse et al., 2024) of children aged 1.5–11 years conducted in the United States, Canada, and the Netherlands between 1999 and 2022, found that compared to pregnancies with no cannabis exposure, children who were exposed to cannabis during pregnancy had a significantly increased risk of ADHD diagnosis (β =0.39 [95 %CI: 0.20 – 0.58]). The findings remained significant after analysis by subgroups adjusting for maternal tobacco smoking, alcohol intake, and trimester of exposure, or whether diagnostic or screening tools were used. For example, a subgroup analysis employing screening tools found an increased risk for ADHD diagnosis (β =0.54 [95 %CI: 0.26 – 0.82]). Similarly, a meta-analysis of six studies on children aged between 2 and 14 years old found that children who were exposed to cannabis in utero had 1.13 times higher odds [95 %CI:1.01–1.26] of ADHD diagnosis compared to children who were not exposed (Bassalov et al., 2024). Both meta-analyses suggest an association between prenatal cannabis exposure and the occurrence of ADHD in offspring, with exposed children having an increased risk of developing ADHD compared to unexposed. The meta-analysis by Tadesse et al. (2024) primarily included studies assessed as high quality according to the Newcastle Ottawa Quality Assessment Scale (NOS). Three of the six studies analyzed by Bassalov et al. (2024) measured exposure based on the mother’s self-report only, while the remaining were assessed using urine screening and prenatal records. Despite these measurement differences, considering that 8 of these studies by Tadesse et al. (2024) were considered high quality, and 3 of the studies by Bassalov et al. (2024) used definitive measures for cannabis exposure, these findings generally provide a solid basis for the evidence presented

The association between the development of autism spectrum disorder (ASD) and cannabis use during pregnancy is less studied. A meta-analysis of four studies (Tadesse et al., 2024) conducted in the United States, Canada, and Australia between 2020 and 2023 in children aged 2–5 years, found that compared to children who were not prenatally exposed to cannabis, children who were exposed had a significantly increased risk of ASD (RR = 1.30 [95 %CI: 1.03 – 1.64]). A separate meta-analysis of five studies of children aged 2–20 years found no association between exposure to cannabis during pregnancy and the development of ASD in offspring (Bassalov et al., 2024). The findings remained non-significant even after a sensitivity analysis omitted an outlier group of children aged 19–20. Similarly, another retrospective cohort study was conducted examining the influence of cannabis exposure in the early stage of pregnancy (8–10 weeks of gestation) on the development of ASD in offspring (Avalos et al., 2024c). After 12–143 months (1–11 years) of follow-up, the authors found no association between children who were exposed to cannabis in early pregnancy and their counterparts who were not exposed with a hazard ratio of 1.05 [95 % CI: 0.84 – 1.32]. Considering the differences between findings, more research is needed to ascertain the impact of prenatal cannabis exposure and the incidence of ASD in offspring.

#### 3.2.2 Mood and emotional disorders

A few studies have examined the effect of in utero cannabis exposure on mental health outcomes with differing results. A meta-analysis examined the impact of the use of cannabis during pregnancy and possible neuropsychiatric outcomes in progeny, including depression and anxiety in children aged 3.2–9 years (Bassalov et al., 2024). An analysis of five studies on anxiety between 1978 and 2016 and three studies on depression between 1982 and 2015, found no significant association between anxiety or depression among children exposed to cannabis during pregnancy compared to children who were not exposed to cannabis use during pregnancy. Results showed a non-significant pooled higher odds of 1.34 [95 %CI: 0.79 – 2.29] and lower odds of 0.72 [95 %CI: 0.11 – 4.57], respectively, for anxiety and depression. Two of the anxiety studies and 4 of the depression studies relied on self-reports.

In contrast, a study conducted among children between ages 9–10 found significant results (Lepow et al., 2024). This study examined the effect of prenatal substance use assessment through self-reports during pregnancy and childhood trauma on offspring neurobiological markers responsible for emotion processing. The substances assessed were a combination of cannabis, cigarettes, and alcohol. Examination of the brain activity of these children showed that compared to children who were not exposed to substance use in pregnancy, children who were exposed to substances during pregnancy had differing brain activity accompanied by behavioral problems reported by their parents, indicating these children were more susceptible to the negative effect of childhood trauma on mental health and brain development additionally indicating the effect of cannabis on offspring when used alongside other substances.

Similarly, a study from a longitudinal perspective cohort of participants enrolled between 2016 and 2018 found significant results (Baranger et al., 2022). With cannabis exposure relying on retrospective reports, the authors found that adolescents exposed to cannabis during pregnancy were more likely to experience psychopathological outcomes including conduct problems, aggression, and rule-breaking behavior. Given the varying outcomes among studies and reliance on self-reported cannabis exposure measures in most studies, more research utilizing more concrete measures of cannabis exposure, such as urine analysis, is necessary to enhance our confidence in the results.

#### 3.2.3 Learning and academic outcomes

The following section describes applied neurodevelopmental outcomes impacting learning and academic performance as assessed in various studies.

##### 3.2.3.1 Diagnosis of learning delays

Numerous studies have explored the effect of cannabis exposure on offspring’s learning delays in various ways. A cohort study of children up to five and a half years old, born between years 2015 and 2019, examined the impact of cannabis exposure —measured through self-report or a positive urine test in early pregnancy (8–10 weeks of gestation)—on neurodevelopmental outcomes. These outcomes included global delays, motor delays, and speech and language disorders assessed using ICD-9 and ICD-10 codes (Avalos et al., 2024b). The results identified no significant association with neurodevelopmental delays including global delays (HR = 1.04; 95 % CI: 0.68–1.59), motor delays (HR = 0.86 = 95 % CI: 0.69–1.06), and speech and language disorders (HR, 0.93; 95 % CI, 0.84–1.03). Aligning with this finding, a study conducted in a cohort of children birthed between the years 1989 and 1992 found no significant association between prenatal cannabis (marijuana) exposure and language deficits (Isik et al., 2023). Employing the Clinical Evaluation of Language Fundamentals (CELF) scores, results showed that compared to kids with no cannabis (marijuana) exposure in utero, children exposed to marijuana during pregnancy had no significant difference in total calculated CELF score −0.33 [95 % CI: −4.71–4.05] at age 10. However, cannabis exposure from previous generations may appear different in the current generation and thus not generalizable.

Conversely, a study of 207 pregnant women enrolled from 2007 to 2015, assessed their children’s development up to 12 months of follow-up using the Mullen Scales of Early Learning (MSEL). Findings showed that infants exposed to cannabis in utero assessed through self-report performed better, having higher scores for gross motor, expressive, and receptive language at 12 months compared to unexposed infants (Talavera-Barber et al., 2023). However, in total contrast to previous studies a study that investigated the effect of cannabis on children, in a cohort of 64 children requiring admission to the NICU from 2018 to 2019, using the Bayley Scales of Infant and Toddler Development (Bayley-III) found a significant negative association (Jarque et al., 2024). This study examined the impact of any substance use during pregnancy measured through biomarkers present in meconium samples within 24 h of birth, confirmed using gas chromatography/mass spectrometry. The authors examined the effect of exposure on motor, cognitive, and language development at 3 years. Results showed that children exposed to any substance during pregnancy had an increased risk of language deficits compared to unexposed children (92.5 [83.8–99.3] vs. 97.0 [89.0–100.0]; p = 0.044], with effects more common and severe among male offspring exposed to cannabis. Additionally, findings showed that children exposed to cannabis had significantly lower composite motor skills scores and worse developmental delays. Further research is necessary to elucidate the effect of cannabis use during pregnancy on speech, language, motor, and global-related delays, using a representative population and extended follow-up periods.

##### 3.2.3.2 School Performance

There is limited research assessing the impact of in utero cannabis exposure and school performance in school-aged children. Although a recent review suggested that exposure to cannabis in pregnancy is associated with poorer academic performance among exposed children compared to unexposed children (Gerede et al., 2024), the studies included in the review did not directly investigate the effect of prenatal cannabis exposure on offspring’s school performance. Furthermore, one study assessed the effect on adolescents, and two others focused on university-level students, with cannabis exposure in these studies evaluated based on the participants' usage rather than exposure during pregnancy. The lack of evidence in this area indicates a need for more research to understand the impact of in utero and early childhood exposure to cannabis on the academic performance of school-aged children.

### 3.3 Cannabis associated dose response relationship

Cannabis has been studied in adolescents for its impact on physical and mental health. The primary focus in larger, longitudinal studies has been on the relationship between the concentration of THC and cannabidiol (CBD) with developmental outcomes and mental health. A recent study of 2262 adolescents between the ages of 9–15 measured THC and CBD concentrations in hair samples over 4 years and evaluated physical and mental health factors (Aks et al., 2025). The study determined that the effects of THC and CBD were different by sex and often presented in opposing ways. THC concentrations were positively correlated with physical and strength exercise in the full sample while CBD concentrations were negatively correlated with strength exercise and internalizing symptoms (somatic complaints, depressed, anxious, withdrawn). In male participants (N = 754, p = .03), there was a positive correlation between sleep duration and CBD concentration whereas female respondents (N = 666, p ≤ 0.001) presented with a negative correlation between THC concentration and sleep duration. For males (N = 793, p = .03), there was a negative correlation between CBD concentration and externalizing symptoms (rule-breaking and aggressive behavior). This correlation was not significant for female respondents. The authors concluded that moderate to high frequent consumption of cannabis does impact mental health differentially based on sex assigned at birth. Male adolescents present with more aggressive and risk-taking behavior at higher THC concentrations. CBD, on the contrary, appears to have a calming, anti-depressant, and anxiolytic effect which is more pronounced in females and may facilitate sleep in both sexes (Aks et al., 2025). The study did not conclude that previous cannabis exposure would lead to either behavioral or mental health changes but rather only explored the impact of current cannabis use.

In a related study from the same research group, neurocognition was evaluated in a subset of adolescent cannabis users aged 13–14 years (N = 246) and correlated with 11-nor-9-carboxy-tetrahydrocannabinol (THCCOOH), the primary inactive metabolite of THC (Wade et al., 2024). While cannabis users (N = 123) overall presented with similar neurocognition patterns compared to the control, there was a negative correlation between picture vocabulary task (p = .03) and Flanker inhibitory control task performance (p = .04) with hair concentrations of THCCOOH. This indicates that moderate to high use of cannabis may lead to short-term memory decreases as well as more aggressive behavior. There is clear indication that there is a dose-dependent relationship between cannabis use in adolescents and at least transient deficits in neurocognition from these two studies.

In a retrospective cohort study among pregnant individuals (6.2 % or 22,624 cannabis exposed neonates out of N = 364,924), cannabis use was associated with a higher risk of pre-term birth and lower birthweight (Avalos et al., 2024a). The study did find a positive correlation between increased risk of pre-term birth (OR: 1.06) and low birthweight (OR: 1.20) and frequency of cannabis use during pregnancy although the authors caution that other factors such as pre-existing health conditions may limit the interpretive value. Furthermore, less than half of cannabis-using pregnant individuals disclosed how frequently they used cannabis during their pregnancy.

Few studies to date have established the pharmacokinetics of THC and CBD during pregnancy and the specific distribution into fetal tissue across the placenta. It is clear that THC and other cannabinoids do cross the placenta and impact development in utero (Grant et al., 2018). A study design proposes to correlate frequency of cannabis use with neonatal outcomes to establish a risk threshold (Robinson et al., 2024). While less than monthly use of cannabis during pregnancy may not increase the risk for adverse neonatal outcomes, with increasing potency of cannabis products and long half-lives for THC and other cannabinoid metabolites, exposure may not need to be frequent to impact neonatal development. More research is needed to conclusively link specific cannabis frequency and dose exposure to neonatal and early childhood development outcomes.

## 4 Discussion

This review aimed to synthesize existing clinical knowledge on neurodevelopmental and related functional outcomes in progeny exposed to cannabis *in utero* or through secondary (“second-hand”) exposure during early childhood. Neurodevelopmental outcomes following *in utero* exposures have been assessed in various ways; however, the effect of secondary exposure is largely understudied. Research examining prenatal cannabis exposure consistently indicates a potential link with the development of ADHD, and no significant association with anxiety and depression. In contrast, findings related to ASD, memory, Attention, and learning delays are mixed and remain inconclusive. Additionally, the effect of prenatal exposure on academic performance has received limited attention and is not well understood. While it is unclear whether cannabis use during pregnancy presents with a dose-outcome relationship, i.e. whether higher doses or more frequent use of cannabis is correlated with lower birthweight or developmental outcomes, there is a clear correlation between use of cannabis during adolescence with benefits (e.g., increased exercise performance) and adverse outcomes such as sleep quality being negatively correlated with THC concentrations or impacted executive and memory functioning.

There are several research method limitations as well as persistent practical research implementation barriers (namely, regulatory) that need to be addressed for us to gain a clearer understanding of the influence of cannabis on neurodevelopment. In terms of limitations with current research methods, the absence of standardized measures of cognition makes it challenging to systematically compile and compare findings across studies that assess cognition-relevant outcomes. This was particularly evident in the systematic review conducted by Torres et al. (2020) who systematically reviewed 1001 papers. The authors noted that one of the reasons why they did not conduct a meta-analysis was because of the heterogeneity in cognitive outcomes measurements, where a total of 36 different outcome measures were identified. Relatedly, inconsistencies in how cannabis exposure was quantified further impeded their ability to conduct a meta-analysis. These challenges underscore the need for standardized methods to measure both exposure and outcome in cannabis and cognitive outcome-related studies.

Observational research on cannabis use has been reliant on self-reported cannabis use measures, which generate imprecise or limited information (Lepow et al., 2024, Talavera-Barber et al., 2023, Torres et al., 2020, Willford et al., 2021). The most frequently used self-reported measures typically rely on recency of use (e.g., episodes of use within the past 24 h, past week, past month, past year, lifetime use), or use subjective assessments of use ‘amount’ (e.g., ‘heavy’ use). Such measures cannot assess precise dosing and cumulative exposures. Integrating self-report measures with confirmatory lab assessments can improve reliability and provide more accurate exposure measurements. These approaches would bring research closer to achieving reliable measures of cannabis exposures, but there are still challenges in defining a cannabis ‘dose’ that are more fundamental measurement issues to tackle. For most outcomes assessed, there is no clear cannabis exposure threshold definitively associated with adverse effects. When not investigator-administered (such as in observational research), it is challenging to quantify precisely the cannabis exposure as experienced by the participant (Jugl et al., 2021, Volkow and Weiss, 2020).

Future systematic reviews and meta-analyses need to consider the generational variations in cannabis use and concentrations, which are consistently evolving (Chaput et al., 2020). Therefore, accounting for these changes in systematic reviews and meta-analyses can better reflect the effect of cannabis and THC within specific timeframes. For example, in the meta-analysis conducted by Thompson et al. (2023), although the overall finding showed no effect, it was interesting to find that studies that had no effect were published after 2011, and those that found an effect were published before 2003. Future studies should consider the increasing THC concentrations and composition of cannabis products.

Although randomized clinical trials are considered the gold standard in epidemiology studies to establish association between exposures and outcomes (Bothwell et al., 2016, Marcaccio and Schermerhorn, 2024), there are major regulatory barriers to conducting research regarding maternal cannabis exposures in progeny, as well as ethical and legal implications, that make this challenging to overcome. In that regard, most of the studies included in this review were found to be cohort, case-control, and cross-sectional studies (Tadesse et al., 2024, Thompson et al., 2023). Furthermore, trials of cannabis use are often limited to pre-clinical context and applications (and may solely evaluate the effects of THC), and doses administered during such trials are often not reflective of real-world dosing and use patterns (or even in the route of administration) (English et al., 2024, National Academies of Sciences et al., 2017).

Confounding remains a persistent and significant challenge. Many of the reviewed studies did not adequately address key confounding factors, including but not limited to the concomitant use of other substances (Torres et al., 2020), the source or type of cannabis used (D’Amico et al., 2020), general and cannabis specific risk perceptions, cannabis-related and other risk behaviors, health literacy, and health behaviors unrelated to substance use, such as exercise, nutrition, and sleep (Cernigliaro et al., 2024, McDonald et al., 2021, Sejbuk et al., 2022).

### 4.1 Implications

To enhance the quality of evidence and clarify unresolved and unexplored research domains noted above, several research approaches can be adopted or modified. Researchers should consider re-assessing cannabis use measures at every interval in which the outcome is assessed, rather than relying on carry-forward observations of the exposures. Additionally, there is a need to standardize the operationalization of cognition measures and employ validated tools to assess the outcome measure. This will allow possible systematic cumulation and synthesis of overall findings in a standardized and objective manner by researchers across various contexts. Furthermore, employing measures that use both self-reports confirmed with laboratory measures can improve the precision and accuracy of cannabis exposure measures. When conducting meta-analysis, it is important to consider the period within which studies were published, to allow for adequate generalizability within an appropriate period and context. Finally, where possible, longer follow-up studies should be applied as neurodevelopmental effects are less likely to be observable within short periods (Castelli et al., 2024).

## 5 Conclusion

This review can help clinicians and patients to understand the current available knowledge from clinical literature on neurodevelopmental and related functional outcomes in progeny who have been exposed to cannabis *in utero* or who have experienced secondary (“second-hand”) exposure to cannabis in early childhood. Gaps remain in our understanding of neurodevelopmental effects on the progeny from *in utero* cannabis exposures. These are largely driven by research regulatory barriers in addition to limitations on current measurement strategies of the cannabis exposure that rely on lower information ‘recency’ of exposure measures rather than precisely quantifying cannabis exposures.

## CRediT authorship contribution statement

**Oliver Grundmann:** Writing – review & editing, Writing – original draft, Formal analysis, Data curation, Conceptualization. **Chidimma Doris Azubuike:** Writing – review & editing, Writing – original draft, Formal analysis, Data curation. **Amie J Goodin:** Writing – review & editing, Supervision, Conceptualization.

## Funding source

This research did not receive any specific grant from funding agencies in the public, commercial, or not-for-profit sectors.

## Declaration of Competing Interest

I have nothing to declare.
